# Pregnant Women With Gestational Diabetes Mellitus and High Consumption of Tryptophan‐Rich Foods Show Increased Levels of 6‐Sulfatoxymelatonin in Urine

**DOI:** 10.1155/jp/4762066

**Published:** 2026-07-10

**Authors:** Daiane Bonella Coltro, Jorge de Oliveira Mateus, Igor de Oliveira Ferreira, Márcia Inês Goettert, Ana Paula Costella, Guilherme Liberato da Silva, Iraci L. S. Torres, André Anjos da Silva, Gabriela Laste

**Affiliations:** ^1^ Medical Student-Universidade do Vale do Taquari-Univates, Lajeado, Rio Grande do Sul, Brazil; ^2^ University of Tübingen, Tübingen, Germany, uni-tuebingen.de; ^3^ Post Graduate Program in Medical Science-PPGCM-Universidade do Vale do Taquari-Univates, Lajeado, Rio Grande do Sul, Brazil; ^4^ Laboratory of Pain Pharmacology and Neuromodulation: Preclinical Investigations-Hospital de Clínicas de Porto Alegre, Porto Alegre, Rio Grande do Sul, Brazil

**Keywords:** 6-sulfatoxymelatonin, gestational diabetes mellitus (GDM), TNF-*α*, tryptophan

## Abstract

**Background:**

Gestational diabetes mellitus (GDM) is associated with metabolic and inflammatory alterations that may influence melatonin metabolism. This study evaluated associations between dietary intake of tryptophan‐rich foods, urinary 6‐sulfatoxymelatonin, and serum tumor necrosis factor‐alpha (TNF‐*α*) levels in pregnant women with GDM.

**Methods:**

A cross‐sectional study included 34 women with GDM and 18 with normal‐risk pregnancies. Dietary habits were assessed using a validated food frequency questionnaire (FFQ). Serum TNF‐*α* and first‐morning urinary 6‐sulfatoxymelatonin levels were measured by ELISA. Associations were evaluated using generalized linear models (GLMs), adjusting for maternal age, body mass index (BMI), gestational age, and sleep quality.

**Results:**

Women with GDM reported a significantly higher frequency of consumption of tryptophan‐rich foods, particularly milk and dairy products (*p* < 0.01) and fruits (*p* < 0.05). The GDM group also exhibited higher urinary 6‐sulfatoxymelatonin (*p* = 0.002) and serum TNF‐*α* levels (*p* = 0.023). In multivariable models, GDM remained significantly associated with higher urinary 6‐sulfatoxymelatonin levels, whereas TNF‐*α* concentrations were primarily associated with maternal characteristics and interaction effects.

**Conclusions:**

GDM was associated with higher urinary 6‐sulfatoxymelatonin and TNF‐*α* levels despite greater reported intake of tryptophan‐rich foods. These findings highlight associations between dietary precursors, melatonin metabolism, and inflammatory markers in hyperglycemic pregnancy and underscore the need for longitudinal studies to clarify underlying mechanisms.

## 1. Introduction

Pregnancy is a physiological condition characterized by profound metabolic, hormonal, and immunological adaptations. Approximately 10%–15% of pregnancies are classified as high‐risk due to the development or worsening of maternal conditions that may compromise both maternal and fetal health [1]. Gestational diabetes mellitus (GDM) is among the most common metabolic complications of pregnancy and is characterized by insulin resistance, low‐grade inflammation, and increased risk of adverse obstetric outcomes [2].

Exposure to light at night and disrupted sleep patterns have been associated with metabolic disturbances and an increased risk of pregnancy complications, including GDM. Recent evidence indicates that both short and long sleep duration are associated with an increased risk of GDM [3]. These findings underscore the importance of circadian regulation in metabolic homeostasis during pregnancy.

Melatonin, a neuroendocrine hormone secreted primarily by the pineal gland, plays a central role in circadian rhythm regulation. Its secretion peaks during the night, typically 3–5 h postsleep onset, and declines with light exposure [4, 5]. Melatonin biosynthesis depends on the availability of tryptophan, an essential amino acid obtained through diet, which is converted into serotonin and subsequently into melatonin [6]. Dietary intake of tryptophan‐rich foods, including dairy products and various plant‐based foods such as apples, barley, beans, cucumber, grapes, lupine, maize, potatoes, rice, and tomatoes, may influence melatonin synthesis by providing precursor substrates [7–9].

Altered melatonin secretion has been associated with metabolic disorders, including obesity, metabolic syndrome, and insulin resistance [10]. In nonpregnant populations, lower nocturnal melatonin excretion, measured as urinary 6‐sulfatoxymelatonin, has been associated with increased risk of Type 2 diabetes [11]. Experimental studies in animal models suggest that melatonin may influence insulin secretion and oxidative stress pathways [12], although translational evidence in human pregnancy remains heterogeneous [13, 14].

In the context of GDM, previous clinical investigations have reported alterations in melatonin metabolism and its interaction with inflammatory markers. A recent study evaluating melatonin, urinary 6‐sulfatoxymelatonin, and cytokines in women with GDM demonstrated differences in melatonin‐related parameters compared with normoglycemic pregnancies [15]. Furthermore, inflammatory processes play a central role in GDM pathophysiology. Elevated circulating levels of tumor necrosis factor‐alpha (TNF‐*α*) have been consistently reported in women with GDM and are associated with insulin resistance and adverse metabolic outcomes [16, 17]. Meta‐analytic evidence confirms that higher TNF‐*α* levels are associated with increased odds of developing GDM [18], and recent studies reinforce TNF‐*α* as a relevant inflammatory marker in this condition [18, 19].

Clinical studies have also investigated melatonin levels and supplementation in other high‐risk pregnancies, including preeclampsia and fetal growth restriction [20]. However, the relationship between dietary intake of melatonin precursors, urinary melatonin metabolites, and inflammatory markers in women with GDM has not been systematically evaluated. Specifically, whether higher intake of tryptophan‐rich foods is associated with normalization of melatonin metabolism and inflammatory status in GDM remains unclear

Therefore, the present study is aimed at assessing urinary 6‐sulfatoxymelatonin levels, a surrogate marker of nocturnal melatonin secretion, and serum TNF‐*α* concentrations in women with GDM compared with normal‐risk pregnancies. We hypothesized that women with GDM would exhibit altered melatonin metabolism and higher inflammatory marker levels, and that dietary intake of tryptophan‐rich foods could be associated with variations in melatonin metabolite levels.

## 2. Methods

A cross‐sectional quantitative study was conducted in the Vale do Taquari region, located in the central region of the state of Rio Grande do Sul (RS), Brazil. This region comprises 37 municipalities under the jurisdiction of the 16th Regional Health Coordination, with a total population of 325,412 inhabitants, according to the 2010 census (16^a^ CRS, 2021). This research was approved by the Research Ethics Committee of Univates under protocol number CAAE: 38885320.0.0000.5310, with approval number 5.480.548, and it complied with Resolution no. 466/2012 of the Ministry of Health. Written informed consent was obtained from all participants prior to enrollment in the study. The study was carried out at the High‐Risk Pregnancy Outpatient Clinic (AGAR), a referral center for the 16th Regional Health Coordination, as well as at the Maternal and Child Care Center (Cami) and the Central Health Post, all located within the municipality of Estrela, RS.

Inclusion criteria for GDM were based on guidelines established by the Pan American Health Organization, the Brazilian Ministry of Health, the Brazilian Federation of Gynecology and Obstetrics Associations, the Brazilian Diabetes Society [21], the International Diabetes Federation [22], the American Diabetes Association [23], and World Health Organization [24]. Pregnant women were included in the GDM group if they met at least one of the following criteria: Fasting plasma glucose between ≥ 92 and ≤ 125 mg/dL during prenatal screening; or at least one abnormal value in the 75 g oral glucose tolerance test (OGTT), performed between 24 and 28 weeks of gestation—defined as fasting glucose ≥ 92 mg/dL, 1‐hour glucose ≥ 180 mg/dL, and 2‐hour glucose ≥ 153 mg/dL. Women with a previous diagnosis of diabetes mellitus prior to pregnancy or those using antidiabetic medications before enrollment were excluded.

The control group consisted of pregnant women with normal‐risk pregnancies, without a diagnosis of GDM based on the same screening criteria. Control participants were not using medications other than ferrous sulfate or folic acid.

Exclusion criteria for both groups included alcohol abuse, illicit drug use, use of analgesic, anti‐inflammatory or central nervous system depressant medications (including anticonvulsants, antidepressants, mood stabilizers, anxiolytics, and antipsychotics), twin pregnancy, neurological disease, inflammatory disease, infectious disease, other high‐risk pregnancy complications, and age under 18 years.

Data was collected from March 2021 to September 2022. Information obtained from medical records of participating pregnant women included admission date, birthdate, weight, height, marital status, education, occupation, functional status, work shift, last menstrual period date, gestational age, number of pregnancies, parity, number of miscarriages, gestational age at time of diagnosis, current use of medication, alcohol consumption, and examination results. Additionally, sociodemographic questionnaires with descriptions of previous illnesses and medication use, Beck Depression Inventory, the food frequency questionnaire (FFQ), and the Pittsburgh Sleep Quality Index (PSQI) were administered.

The Beck Depression Inventory is a self‐report scale consisting of 21 items used to assess the intensity of depressive symptoms. Dietary intake was assessed using a validated FFQ designed to evaluate the frequency of consumption across specific food groups. The instrument used categorical response options and did not provide quantitative estimates of daily nutrient intake (e.g., grams or milligrams per day) [25]. Consequently, absolute tryptophan intake could not be calculated, and the analysis focused on reported consumption patterns of tryptophan‐rich foods rather than quantitative nutrient estimation. The PSQI is a self‐administered questionnaire (Likert scale from 0 to 3) developed by the University of Pittsburgh, Department of Psychiatry (Pittsburgh, Pennsylvania, United States) to evaluate sleep quality. For all items, higher scores indicate higher sleep disruption.

A 10 mL blood aliquot was collected from each individual. The blood was then centrifuged at 4500 rpm for 10 min at 6°C, and the supernatants of the samples were stored in an ultrafreezer at −80°C until subsequent analysis.

Urine samples for 6‐sulfatoxymelatonin measurement were collected in the morning (first morning urine) in 350 mL containers. Previous studies have indicated that urinary excretion of 6‐sulfatoxymelatonin in the first morning urine accurately reflects melatonin levels in plasma during the previous night [26]. A 40 mL aliquot of urine was centrifuged at 4000 rpm for 5 min at 6°C, and the supernatants were immediately stored in an ultrafreezer at −80°C.

The serum levels of TNF‐*α* (Invitrogen 88‐7344‐88) and urinary levels of 6‐sulfatoxymelatonin (Elabscience E‐EL‐H1726) were analyzed using commercial ELISA kits. Samples were quantified using a standard curve, and optical density was measured at 490 nm.

Following data collection in Excel, two researchers reviewed and corrected inconsistencies. Analyses were performed using available data for each variable. Participants with incomplete biological samples for a given biomarker were excluded from that specific analysis. No data imputation procedures were applied. Statistical analysis was performed using SPSS Statistics software (Version 20.0.0). The Kolmogorov–Smirnov and Shapiro–Wilk tests were used to assess data normality. Nonparametric continuous samples were analyzed using the Mann–Whitney *U* test. Descriptive data were expressed as median and interquartile range. The number and percentage were expressed as *n* (%), and nominal and ordinal categorical variables were analyzed using the chi‐squared (*χ* ^2^) and Fisher′s exact tests. The dependent variables for this study were Melatonin and TNF‐*α* levels. Therefore, to evaluate the effects of independent variables on this outcome, generalized linear models (GLMs) with a gamma distribution and log function were used, as the analyzed variables did not have a linear distribution. Post‐hoc analyses employed Bonferroni adjustments. The lowest Akaike′s Information Criterion (AIC) value was used to determine the most appropriate model.

A priori sample size estimation was performed using BioEstat 5.3 software based on previous studies [27–30]. The calculation indicated that 30 participants per group (total *n* = 60) would be required to achieve 90% statistical power with *α* = 0.05.

A total of 79 pregnant women were initially assessed for eligibility between March 2021 and September 2022. After application of inclusion and exclusion criteria, 52 participants were included in the final sample in the GDM group and 18 in the normal‐risk pregnancy group. Not all biological samples were available for laboratory analysis. Due to incomplete biological sample collection and sample losses, the final number of participants included in biomarker analyses was 38 for serum TNF‐*α* (GDM *n* = 26; NRP *n* = 12) and 32 for urinary 6‐sulfatoxymelatonin (GDM *n* = 21; NRP *n* = 11).

**Figure 1:**
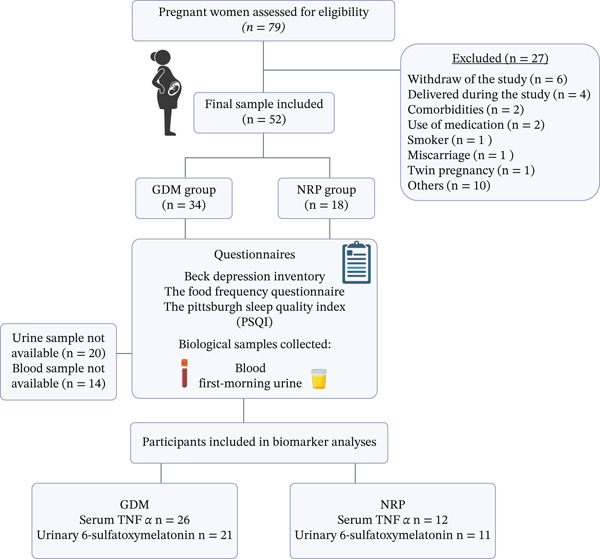
Flow diagram of participant recruitment, eligibility assessment, sample collection, and biomarker analyses in pregnant women with gestational diabetes mellitus (GDM) and normal‐risk pregnancies (NRP).

The participant flow, including exclusions and sample losses, is detailed in Figure 1.

A *p* value of < 0.05 was considered statistically significant for all analyses.

## 3. Results

Sociodemographic and clinical characteristics related to pregnancy are summarized in Table 1. Women in the GDM group were slightly older than those with normal‐risk pregnancies (NRP). Mean gestational age at assessment was 21.8 weeks (range: 9–37) in the GDM group and 25.5 weeks (range: 6–37) in the NRP group. Reproductive history, including number of pregnancies, parity, and previous miscarriages, was comparable between groups.

**Table 1:** Sociodemographic and clinical characteristics related to pregnancy for women with normal risk pregnancies (NRP) and pregnant women with gestational diabetes mellitus (GDM) (*n* = 52).

	NRP *n* = 18				GDM *n* = 34			
	m	sd	*n*	%	m	sd	*n*	%
**Age**	25.8	5.3			28.1	6.0		
**Gestational age** ^∗^	25.5	8.5			21.8	7.8		
**Number of pregnancies**	1.8	1.1			2.1	1.1		
**Parity**	0.7	1.0			1.0	1.2		
**Number of miscarriages**	0.1	0.4			0.2	0.5		
**Pittsburgh Sleep Quality Index (SQI)**	14.3	8.2			14.8	7.2		
**Beck′s Depression Inventory**	14.0	9.7			10.8	7.5		
**Alcohol consumption**								
On weekends			4	20			3	15
Rarely			4	20			8	45
No consumption			10	60			23	40
**Education**								
Incomplete elementary education			1	5.6			4	11.8
Complete elementary education			0	0			2	5.8
Incomplete high school education			4	22.2			4	11.8
Complete high school education			10	55.6			13	38.2
Incomplete higher education			1	5.6			2	5.8
Complete higher education			0	0			7	20.6
Postgraduate education			1	5.6			0	0
Not reported			1	5.6			2	5.8
**Civil status (n = 33)**								
With partner			13	72.2			31	91.2
Single			5	27.8			2	0.8
**Functional status**								
Inactive			2	11.1			2	5.9
Active			15	83.3			26	76.5
Welfare			1	5.6			6	17.6
**Profession**								
Housekeeper			5	27.8			5	14.7
Commerce			7	38.9			13	38.2
Others			6	33.3			16	47.1
**Work shift**								
Daytime			14	77.8			27	79.4
Nighttime			3	16.7			5	14.7
Not reported			1	5.5			2	5.9
**Gestational BMI (n = 32)**								
Underweight			2	11.1			3	8.8
Normal weight			6	33.3			6	17.6
Overweight			7	38.9			10	29.4
Obesity			2	16.7			13	44.2

*Note:*  ^∗^ denotes in weeks.

Abbreviation: m, mean; sd, standard deviation; SQI, Sleep Quality Index.

All participants reported living with a partner. Educational attainment differed modestly between groups, with 55.6% of women in the NRP group and 38.2% in the GDM group having completed high school. Most participants in both groups worked daytime shifts (GDM: 79.4%; NRP: 77.8%), predominantly in commerce or domestic services (Table 1).

Obesity (BMI ≥ 30 kg/m2) was more frequent in the GDM group (44.2%) compared with the NRP group (16.7%). Regarding alcohol consumption prior to pregnancy, approximately 60% of women in the GDM group and 40% in the NRP group reported some degree of intake.

Sleep quality was assessed using the PSQI. Mean PSQI scores were 14.3 ± 8.2 in the NRP group and 14.9 ± 7.2 in the GDM group, indicating overall poor sleep quality in both groups. Depressive symptoms, evaluated using the Beck Depression Inventory, yielded mean scores of 14.0 ± 9.7 in the NRP group and 10.8 ± 7.5 in the GDM group, corresponding to mild to moderate depressive symptomatology.

Dietary intake of tryptophan‐rich foods is presented in Table 2. Women with GDM reported significantly higher consumption of milk and dairy products (*p* < 0.01) as well as fruits (*p* < 0.05) compared with the NRP group. No significant differences were observed for other tryptophan‐rich food categories (*p* > 0.05 for all comparisons).

**Table 2:** Consumption of foods with significant levels of tryptophan by pregnant women with normal risk pregnancies (NRP) and pregnant women with gestational diabetes mellitus (GDM) analyzed using a food frequency questionnaire (FFQ) (*n* = 52).

	NRP (*n* = 18)		GDM (*n* = 34)		Total		*p*∗
	*n*	%	*n*	%	*n*	%	
Consumption of milk and dairy products							
Yes	12	66.7%	32	94.1%	44	84.6%	0.001
Rare	6	33.3%	0	0.0%	6	11.5%	
No	0	0.0%	2	5.9%	2	3.8%	
Consumption of olive oil							
Yes	5	27.8%	12	35.3%	17	32.7%	0.45
Rare	2	11.1%	1	2.9%	3	5.8%	
No	11	61.1%	21	61.8%	32	61.5%	
Consumption of vegetables and legumes							
Yes	13	72.2%	32	94.1%	45	86.5%	0.072
Rare	4	22.2%	2	5.9%	6	11.5%	
No	1	5.6%	0	0%	1	1.9%	
Consumption of fruits							
Yes	15	83.3%	34	100%	49	94.2%	0.04
Rare	2	11.1%	0	0%	2	3.8%	
No	1	1%	0	0%	1	1.9%	
Consumption of beans							
Yes	17	94.4%	31	91.2%	48	92.3%	0.76
Rare	1	5.6%	2	5.9%	3	5.8%	
No	0	0.0%	1	2.9%	1	1.9%	
Consumption of cattle/pork/poultry meats							
Yes	14	77.8%	33	97.1%	47	90.4%	0.07
Rare	3	16.7%	1	2.9%	4	7.7%	
No	1	5.6%	0	0.0%	1	1.9%	
Consumption of eggs							
Yes	12	66.7%	24	70.6%	36	69.2%	0.95
Rare	3	16.7%	5	14.7%	8	15.4%	
No	3	16.7%	5	14.7%	8	15.4%	
Consumption of fish							
Yes	5	27.8%	8	23.5%	13	25%	0.75
Rare	7	38.9%	11	32.4%	18	34.6%	
No	5	33.3%	15	44.1%	21	40.4%	
Consumption of coffee							
Yes	13	72.2%	22	64.7%	35	67.3%	0.26
Rare	1	5.6%	0	0%	1	1.9%	
No	4	22.2%	12	35.3%	16	30.8%	
Consumption of natural juice							
Yes	6	33.3%	13	38.2%	19	36.5%	0.78
Rare	2	11.1%	2	5.9%	4	7.7%	
No	10	55.6%	19	55.9%	29	55.8%	

*Note:*  ^∗^ denotes chi‐square test (*χ* ^2^). A *p* value < 0.05 was considered statistically significant.

**Figure 2:**
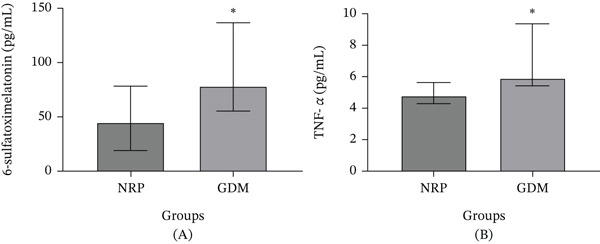
(A) Urinary levels of 6‐sulfatoxymelatonin (*n* = 32) and (B) serum levels of TNF‐*α* (*n* = 38) in women with normal risk pregnancies (NRP) and gestational diabetes mellitus (GDM). *Note:* ∗ denotes the significance level determined by the Mann–Whitney *U* rank test for comparing two distributions *p* < 0.05.

Women with GDM exhibited significantly higher urinary 6‐sulfatoxymelatonin levels (*p* = 0.002) and higher serum TNF‐*α* concentrations (*p* = 0.023) compared with women with NRP (Figure 2).

GLMs were subsequently applied to examine independent associations. GDM was significantly associated with higher urinary 6‐sulfatoxymelatonin levels (*χ* ^2^ = 27.25; *p* < 0.001). Women with GDM exhibited 4.57‐fold higher estimated levels compared with controls (exp[*β*] = 4.57; 95% CI: 2.58–8.08). No significant associations were identified for BMI, maternal age, gestational age, sleep quality, depressive symptoms, or interaction terms (*p* > 0.05).

For TNF‐*α*, the main effect of the group was not statistically significant in the multivariable model. However, maternal age, BMI category, sleep quality, and interaction terms were significantly associated with TNF‐*α* levels. Increasing maternal age was associated with lower TNF‐*α* concentrations (exp[*β*] = 0.95; 95% CI: 0.92–0.98). Using obesity as the reference category, underweight women exhibited higher TNF‐*α* levels (exp[*β*] = 2.10; 95% CI: 1.15–3.83), whereas no significant differences were observed among normal‐weight, overweight, and obese categories. Poorer sleep quality was associated with higher TNF‐*α* levels (exp[*β*] = 1.08; 95% CI: 1.02–1.14; *p* = 0.02). Significant interactions were observed between group and gestational age (exp[*β*] = 1.04; 95% CI: 1.01–1.09; *p* = 0.04) and between group and sleep quality (exp[*β*] = 0.92; 95% CI: 0.86–0.99; *p* = 0.03). Depressive symptoms were not significantly associated with TNF‐*α* levels (*p* > 0.05).

Overall, TNF‐*α* concentrations were associated with covariates and interaction effects rather than with group status alone in adjusted analyses.

## 4. Discussion

The present study demonstrated that women with GDM exhibited higher urinary 6‐sulfatoxymelatonin and serum TNF‐*α* concentrations compared with women with NRP, alongside a higher reported intake of tryptophan‐rich foods. These findings indicate a relationship among inflammation, circadian hormonal control, and tryptophan metabolism in the pathogenesis of GDM.

Urinary 6‐sulfatoxymelatonin is a validated noninvasive surrogate of nocturnal melatonin secretion [31]. Maternal melatonin production increases progressively across gestation, particularly after mid‐pregnancy [32]. Due to the enrollment of participants across a wide gestational range, the physiological variation inherent to pregnancy progression may have influenced metabolite concentrations despite statistical adjustment. This variability must be taken into account when comparing these results with studies conducted within more restricted gestational windows. In this context, the elevated urinary 6‐sulfatoxymelatonin levels observed in women with GDM differ from reports describing reduced circulating melatonin concentrations in hyperglycemic pregnancies [13, 33].

Recent evidence has further explored the interaction between melatonin metabolism and inflammatory pathways in gestational diabetes. França et al. [14] investigated circulating melatonin, urinary melatonin sulfate, inflammatory cytokines, and melatonin receptor expression in pregnant women with GDM, including those with pregnancy‐specific urinary incontinence. The authors reported reduced serum melatonin, decreased urinary melatonin sulfate, and lower IL‐10 levels, accompanied by increased TNF‐*α*, IL‐1*β*, and IL‐8 concentrations and upregulation of MT1 and MT2 receptors. Together, these findings suggest disruption of the melatonin–cytokine axis in GDM, characterized by the coexistence of inflammation, reduced melatonin availability, and increased receptor expression, a pattern consistent with dysregulated melatonin signaling that may contribute to neuromuscular dysfunction of the lower urinary tract. In the present study, elevated urinary 6‐sulfatoxymelatonin levels were observed in women with GDM together with increased TNF‐*α* concentrations. Although urinary incontinence was not evaluated, the consistent elevation of TNF‐*α* in both studies supports the presence of a proinflammatory state in hyperglycemic pregnancies. Differences in urinary melatonin metabolite levels between studies may reflect variations in study design and clinical characteristics of the populations evaluated, particularly because the previous study focused on GDM associated with pregnancy‐specific urinary incontinence, whereas the present analysis examined metabolic and inflammatory markers in women with GDM irrespective of urinary symptoms

Variations among studies may therefore reflect differences in biological matrix selection rather than fundamentally divergent regulatory mechanisms. The coexistence of higher urinary melatonin metabolites and elevated TNF‐*α* levels in women with GDM further contextualizes these findings. Chronic low‐grade inflammation is recognized as a central pathophysiological mechanism of GDM [33]. TNF‐*α*, beyond serving as a marker of systemic inflammation, is strongly associated with insulin resistance by impairing glucose uptake in peripheral tissues [34]. Previous research has also reinforced the role of proinflammatory cytokines in metabolic dysregulation during GDM [34]. Accordingly, the elevated TNF‐*α* levels identified in the present study are consistent with the proinflammatory intrauterine environment described in GDM, which has been associated with maternal metabolic dysregulation and altered placental function [35].

In addition to metabolic and circadian influences, the inflammatory environment observed in GDM is shaped by a complex interplay of genetic, epigenetic, and environmental factors. Evidence suggests that variations in inflammatory signaling pathways may modulate the expression and activity of proinflammatory cytokines, including TNF‐*α* and IL‐6, thereby contributing to insulin resistance and impaired glucose regulation during pregnancy [36]. In this way, it is possible to suggest that the elevated TNF‐*α* concentrations observed in the present study likely reflect broader immune‐metabolic mechanisms involved in the pathogenesis of GDM rather than being attributed only to dietary or circadian factors

Melatonin physiology during pregnancy extends beyond pineal secretion, as extrapineal synthesis occurs in several tissues, including the placenta [5]. Maternal melatonin concentrations rise gradually during gestation and decrease rapidly postdelivery, confirming the placenta as a primary source of endogenous melatonin during pregnancy, with significant placental contribution to circulating melatonin levels [32]. Given that GDM is linked to alterations in placental structure and function, fluctuations in placental melatonin synthesis may have influenced the discrepancies in urine 6‐sulfatoxymelatonin noted in this investigation, independent of dietary tryptophan consumption. At the maternal–fetal interface, melatonin contributes to oxidative balance and mitochondrial regulation [37]. GDM is characterized by activation of placental inflammatory and oxidative pathways [38], whereas melatonin exerts antioxidant and anti‐inflammatory effects in gestational tissues [39]. These placental regulatory processes may influence systemic melatonin metabolite levels. In the present study, elevated urinary 6‐sulfatoxymelatonin levels were observed alongside increased TNF‐*α* concentrations, indicating that alterations in melatonin metabolism occurred within an inflammatory and oxidative context. However, the absence of an inverse association between melatonin metabolites and TNF‐*α* suggests that increased metabolite excretion was not accompanied by measurable attenuation of systemic inflammation. This pattern places melatonin‐related alterations within the proinflammatory environment characteristic of GDM rather than indicating effective compensatory regulation.

Circadian regulation represents another relevant dimension in this context, as circadian rhythms are closely linked to glucose metabolism and insulin sensitivity [18]. Circadian misalignment has also been associated with metabolic dysfunction [40]. In the present study, first‐morning urine collection was used to approximate integrated nocturnal melatonin secretion and to reduce intraindividual variability [25, 41]. Both groups exhibited overall poor sleep quality according to PSQI scores, and no significant difference was observed between them. Consequently, sleep‐related circadian disruption alone is unlikely to explain the variations in melatonin metabolites observed in this population.

The dietary component of the present analysis adds further depth to these findings. Women with GDM reported greater consumption of tryptophan‐rich foods, a biochemical precursor of both serotonin and melatonin [7]. Although increased precursor availability could theoretically influence melatonin synthesis [42], reported dietary intake in the current study does not necessarily correspond to proportional systemic precursor availability. It is important to note that pregnancy is characterized by substantial physiological and metabolic adaptations, including changes in gastrointestinal function, nutrient handling, and maternal metabolism [43]. Furthermore, tryptophan metabolism undergoes marked alterations during gestation, characterized by variations in circulating free tryptophan concentrations and increased flux through the kynurenine pathway to support fetal growth, neurodevelopment, NAD+ synthesis, and maternal–fetal immune tolerance [44]. Collectively, these physiological adaptations may modulate the relationship between dietary tryptophan intake and downstream melatonin synthesis during pregnancy. Moreover, higher intake of tryptophan‐rich foods may reflect dietary counseling following GDM diagnosis, highlighting the dynamic relationship between clinical management and biomarker profiles. Importantly, tryptophan is also metabolized through the kynurenine pathway, which is strongly stimulated by inflammatory signals such as TNF‐*α* [45]. Under proinflammatory conditions, increased indoleamine 2,3‐dioxygenase activity redirects tryptophan metabolism from melatonin synthesis toward kynurenine production [46]. Thus, despite increased dietary tryptophan intake, inflammatory signaling in GDM may alter tryptophan metabolic pathways, shifting the balance between melatonin‐associated protective mechanisms and kynurenine‐mediated immunomodulatory processes.

Taken together, these findings reinforce the central role of inflammation in the pathophysiology of GDM. Although unadjusted analyses demonstrated higher TNF‐*α* levels in women with GDM, multivariable modeling indicated that inflammatory variation was influenced by maternal age, body mass index, and interaction effects. This highlights the multifactorial regulation of immune pathways during pregnancy and emphasizes the necessity of interpreting melatonin‐related alterations within an integrated metabolic framework rather than as isolated endocrine changes.

From a translational perspective, the current results suggest that dietary strategies focused exclusively on increasing tryptophan intake may not be sufficient to significantly modify melatonin metabolism or systemic inflammatory activation in GDM. To fully understand melatonin dynamics during hyperglycemic pregnancy, it is likely necessary to consider endocrine regulation, inflammatory signaling, circadian biology, and metabolic status concurrently [47]. Longitudinal studies that include repeated measurements of hormonal and cytokine, as well as assessment of receptor expression, are essential to elucidate temporal relationships and potential clinical implications.

This study has several strengths. The integrated assessment of urinary 6‐sulfatoxymelatonin, inflammatory markers, and dietary patterns within the same clinical population enhances interpretative consistency. The use of first‐morning urine samples provided a practical estimate of nocturnal melatonin secretion [48], and multivariable modeling enabled adjustment for relevant maternal covariates, strengthening analytical robustness. Several limitations should also be acknowledged. The modest sample size may have limited statistical power. Additionally, the relatively small sample size combined with the inclusion of multiple covariates in the multivariable models may increase the risk of overfitting and limit the stability of parameter estimates. The imbalance between the GDM and normal‐risk pregnancy groups may also have affected the stability and precision of interaction terms in the GLM analyses. Gestational age was not standardized at enrollment, and physiological variation across trimesters may have influenced melatonin metabolite levels [49]. Dietary intake was assessed using frequency‐based categories rather than quantitative nutrient estimation. In addition, blood samples for TNF‐*α* were collected during daytime clinical visits, but exact sampling times were not strictly standardized across participants. Given the known circadian variation of inflammatory markers, this may have introduced additional variability in TNF‐*α* measurements. Finally, biomarkers were measured at a single time point, precluding evaluation of longitudinal endocrine and inflammatory trajectories.

## 5. Conclusion

In conclusion, women with GDM exhibited higher urinary 6‐sulfatoxymelatonin and TNF‐*α* concentrations compared with women with normal‐risk pregnancies, together with greater reported intake of tryptophan‐rich foods. The coexistence of altered melatonin metabolite excretion and elevated inflammatory markers reflects the complex interaction between circadian hormonal regulation and inflammatory processes in hyperglycemic pregnancy. The absence of inflammatory normalization despite higher reported intake of tryptophan‐rich foods suggests that melatonin‐related alterations in GDM are not solely explained by substrate availability. These findings indicate that melatonin metabolites may represent potential indicators of metabolic stress in GDM and highlight the relevance of circadian regulation in metabolic control during pregnancy. Given the cross‐sectional design and exploratory nature of this study, longitudinal and interventional investigations are needed to clarify temporal relationships and better understand the potential role of inflammatory and circadian pathways in the development and progression of GDM.

## Funding

This study was supported by the Universidade do Vale do Taquari, 10901364.

## Conflicts of Interest

The authors declare no conflicts of interest.

## Data Availability

Research data are not shared.
